# Supplementary material for: The long non-coding RNA SNHG4/microRNA-let-7e/KDM3A/p21 pathway is involved in the development of non-small cell lung cancer
Source: Mol Ther Oncolytics. 2020 Dec 25;20:634–45. doi: 10.1016/j.omto.2020.12.010 (PMC7995486; doi:10.1016/j.omto.2020.12.010)
Supplement: Document S1. Figures S1–S3 [file mmc1.pdf]

OMTO, Volume *20*

Supplemental information

The long non-coding RNA SNHG4/microRNA-let-7e/KDM3A/p21 pathway is involved in the development of non-small cell lung cancer

Fan Wang and Qingqing Quan

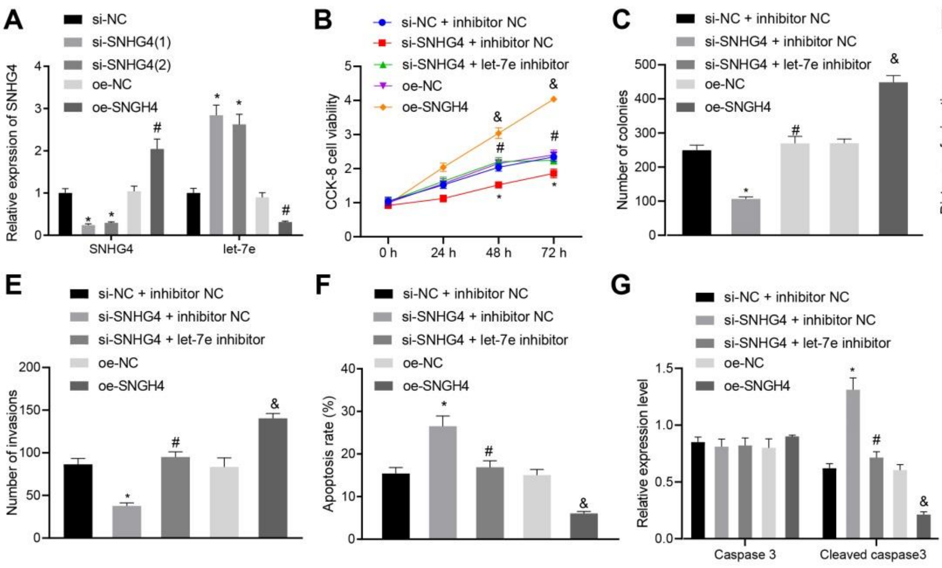

Supplementary Figure 1. LncRNA SNHG4 mediates H1975 cell proliferation, migration, invasion, and apoptosis by binding to miR-let-7e.

A, Expression of lncRNA SNHG4 and miR-let-7e after siRNA knockdown of lncRNA SNHG4 in H1975 cells. B, H1975 cell viability measured by CCK-8 assays. C, Numbers of colonies derived from H1975 cells. D, Scratch wound healing. E, H1975 cells invading from Matrigel-coated upper transwell chambers into lower ones. F, H1975 cell apoptosis determined by Annexin V/PI-labeled flow cytometric analysis. G, Western blot analysis of caspase 3 and cleaved caspase 3 in H1975 cells, normalized to β-actin expression. * (compared with H1975 cells treated with scramble siRNA alone and/or inhibitor NC), # (compared with H1975 cells treated with oe-NC alone or lncRNA SNHG4-specific siRNA plus inhibitor NC) and & (compared with H1975 cells treated with oe-NC alone) indicate $p < 0.05$ by Tukey's test-corrected one-way ANOVA or Bonferroni-corrected repeated measures ANOVA.

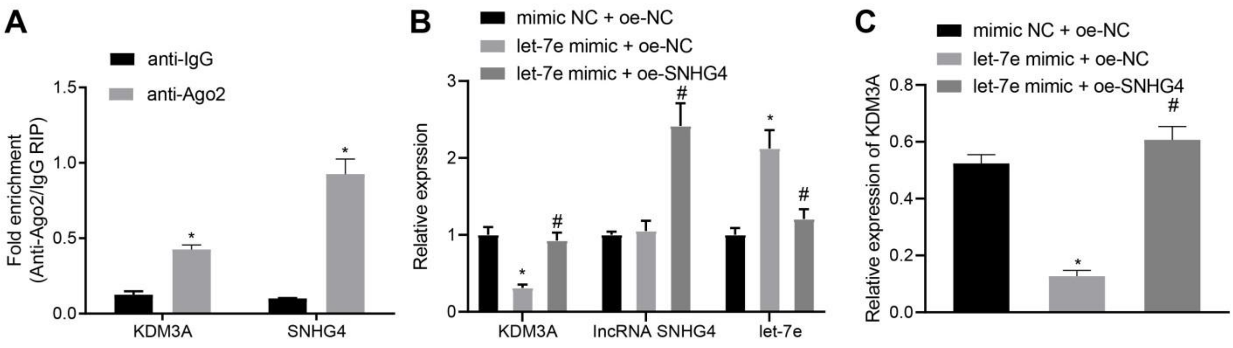

Supplementary Figure 2. LncRNA SNHG4 bound to miR-let-7e and upregulated KDM3A.

A, Anti-Ago2 RIP in H1975 cells transiently overexpressing lncRNA SNHG4. B, The expression of KDM3A was determined by RT-qPCR in H1975 cells. C, Western blot analysis of KDM3A in H1975 cells, normalized to β-actin expression. * (compared with H1975 cells treated with oe-NC with or without mimic NC) and # (compared with H1975 cells treated with miR-let-7e mimic with oe-NC) indicate $p < 0.05$ by unpaired t test or Tukey's test-corrected one-way ANOVA. Pearson Correlation Coefficient was applied to correlation analysis between KDM3A and lncRNA SNHG4.

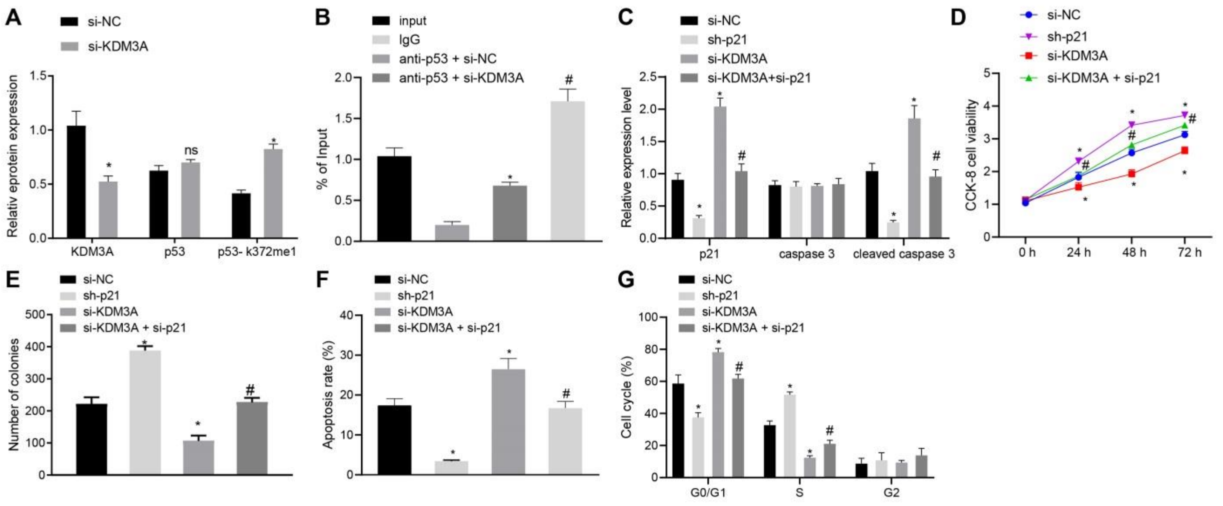

Supplementary Figure 3. KDM3A functioned as an oncogene in NSCLC by inhibiting p21.

A, Western blots and quantification of KDM3A, p53-k372me1, and p53 in H1975 cells, normalized to β-actin expression. B, p21 was immunoprecipitated using p53 antibody relative to IgG by ChIP assays. C, Western blot analysis of p21, cleaved caspase 3, and caspase 3 in H1975 cells, normalized to β-actin expression. D, H1975 cell viability was measured by CCK-8 assays. E, Numbers of colonies derived from H1975 cells. F, H1975 cell apoptosis determined by Annexin V/PI-labeled flow cytometric analysis. G, H1975 cell cycle determined by flow cytometric analysis. * (compared with scramble siRNA or IgG antibody) and # (compared with si-KDM3A or anti-p53 with si-NC) indicate $p < 0.05$ by unpaired t test, Tukey's test-corrected one-way ANOVA or Bonferroni-corrected repeated measures ANOVA.
