# Supplementary material for: The long non-coding RNA SNHG4/microRNA-let-7e/KDM3A/p21 pathway is involved in the development of non-small cell lung cancer
Source: Mol Ther Oncolytics. 2020 Dec 25;20:634–45. doi: 10.1016/j.omto.2020.12.010 (PMC7995486; doi:10.1016/j.omto.2020.12.010)
Supplement: Document S2. Article plus supplemental information [file mmc2.pdf]

Molecular Therapy
Oncolytics
Original Article

The long non-coding RNA SNHG4/microRNA-let-7e/KDM3A/p21 pathway is involved in the development of non-small cell lung cancer

Fan Wang[1] and Qingqing Quan[2]

[1]Department of General Intervention, Linyi People's Hospital, Linyi 276000, P.R. China; [2]Department of Respiratory Medicine, Linyi People's Hospital, Linyi 276000, Shandong Province, P.R. China

Non-small cell lung cancer (NSCLC) is a foremost cause of malignancy-associated mortality globally. Recent studies have emphasized long non-coding RNAs (lncRNAs) as important biomarkers with diagnostic and therapeutic potential in regard to NSCLC. This study aimed to elucidate the functional role of lncRNA small nucleolar RNA host gene 4 (SNHG4) in NSCLC. Initially, 50 paired cancerous and noncancerous tissues were obtained from NSCLC patients. Human NSCLC H1299 cells were assayed to evaluate viability, colony formation, invasion, migration, cycle arrest, and apoptosis via Cell Counting Kit-8 (CCK-8), plate clone formation, and transwell invasion assays, as well as a scratch test and flow cytometry. A dual-luciferase reporter gene assay was used to examine lncRNA SNHG4 binding with miR-let-7e and miR-let-7e binding with lysine demethylase 3A (KDM3A). H1299 cells were xenografted into nude mice. lncRNAs SNHG4 and KDM3A were both upregulated in NSCLC tissues. The knockdown of lncRNA SNHG4 or KDM3A inhibited H1299 cell viability, colony formation, invasion, migration, and cycle progression while inducing apoptosis. lncRNA SNHG4 was found to bind to miR-let-7e that negatively targeted KDM3A. KDM3A inhibited p53-K372me1, thus reducing p21 expression. The NSCLC development was inhibited by downregulating lncRNA SNHG4 in nude mice. Taken together, the key findings of the current study demonstrate a novel lncRNA SNHG4/let-7e/KDM3A/p21 axis in NSCLC, highlighting a promising therapeutic target for NSCLC.

INTRODUCTION

As a distinct cause of cancer-related deaths worldwide, lung cancer is classified into two major histological subtypes: non-small cell lung cancer (NSCLC) and SCLC.[1] Notably, NSCLC accounts for more than 75% of all lung cancer.[2] Despite commendable advances in relationship to lung cancer treatment approaches, patients diagnosed with NSCLC often have a poor prognosis, with an overall 5-year survival rate of less than 16%.[3] Most NSCLC patients are often diagnosed at middle and advanced disease stages, with chemotherapy or combined radiotherapy often used as NSCLC treatment methods.[4,5] The aforementioned treatment approaches are capable of killing tumor cells; however, this also results in damage to normal cells, often consequently leading to significant serious side effects due to poor specificity.[6] Therefore, a more in-depth understanding of the molecular mechanisms underlying NSCLC is of great significance in order to identify more effective prevention strategies and therapeutic methods for NSCLC.

Long non-coding RNAs (lncRNAs) represent a group of non-protein-coding transcripts of more than 200 nt in length.[7] During the past decade, the effects of lncRNA on tumor progression have been demonstrated to contribute to the modulation of cancer cell proliferation, invasion, and metastasis.[8] Several lncRNAs have been implicated in the development of NSCLC.[9] For instance, lncRNA AFAP1-AS1 is capable of facilitating the progression of NSCLC, highlighting its diagnostic and therapeutic values for patients with NSCLC.[10] Interestingly, as a recently discovered lncRNA located in 5q31.2, the small nucleolar RNA host gene 4 (SNHG4), has been implicated in various human cancers.[11] In addition, existing literature has provided evidence verifying the role of lncRNA SNHG4 in lung cancer cells through the regulation of microRNA-98-5p (miR-98-5p),[12] highlighting the critical role of lncRNA SNHG4 in the progression of NSCLC.

MicroRNAs (miRNAs) refer to a group of small non-coding RNAs capable of regulating the expression of their target genes, and they exert oncogenic or tumor-suppressive effects on multiple malignancies.[13,14] miR-let-7 performs as a modulator of cell proliferation, and it may potentially have tumor-inhibiting capabilities.[15] Meanwhile, a prior study has revealed that let-7e expression is closely related to NSCLC.[16] Based on bioinformatics analysis, we subsequently asserted the prediction that lncRNA SNHG4 is a competing endogenous RNA (ceRNA) of miR-let-7, and hence the effect of lncRNA SNHG4 may be related to miR-let-7. Furthermore, lysine demethylase 3A (KDM3A), also known as JMJD1A and JHDM2A, has been reported to remain as an H3K9me1/2 demethylase of the JmjC family and influence the development and progression of certain tumors.[17] Significant evidence confirming the role of KDM3A in lung adenocarcinoma has been

Received 16 August 2020; accepted 21 December 2020;
https://doi.org/10.1016/j.omto.2020.12.010.

Correspondence: Qingqing Quan, Department of Respiratory Medicine, Linyi People's Hospital, No. 27, East Section of Jiefang Road, Lanshan District, Linyi 276000, Shandong Province, P.R. China.
E-mail: doctor2009hiii@163.com

 © 2021 The Authors.
This is an open access article under the CC BY license (http://creativecommons.org/licenses/by/4.0/).

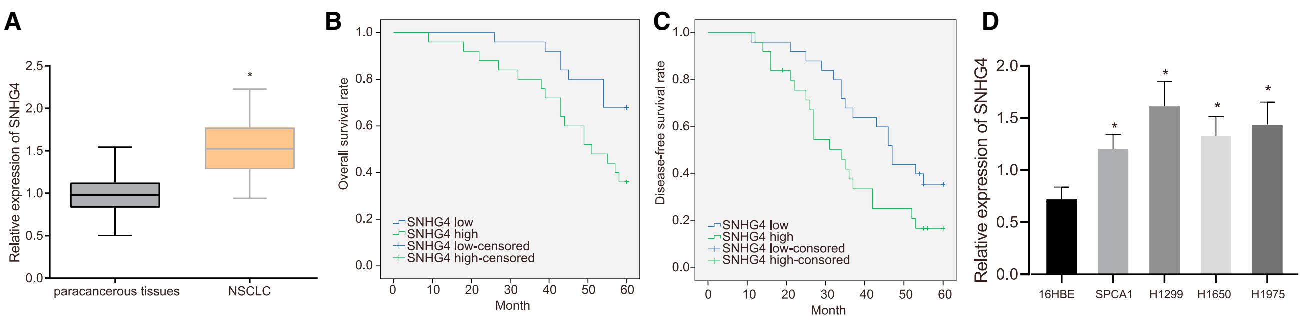

Figure 1. Upregulated lncRNA SNHG4 is associated with NSCLC prognosis

(A) The expression of lncRNA SNHG4 was determined between NSCLC tissues (n = 50) and matched paracancerous lung tissues (n = 50) by qRT-PCR. (B) Kaplan-Meier curves (overall survival) of NSCLC patients according to lncRNA SNHG4 expression. (C) Kaplan-Meier curves (disease-free survival) of NSCLC patients according to lncRNA SNHG4 expression. (D) The expression of lncRNA SNHG4 was determined among H1299, H1650, H1975, SPCA1, and 16HBE cells by qRT-PCR. *p < 0.05 compared with matched noncancerous lung tissues by paired t test or compared with 16HBE cells by Tukey's test-corrected one-way ANOVA.

reported,[18] and KDM3A might be the target gene of miR-let-7 based on bioinformatics data. The current study set out to investigate the effect of lncRNA SNHG4 on the development of NSCLC and subsequently elucidate the associated mechanisms, with the overall objective of identifying novel NSCLC targets or biomarkers.

RESULTS

Upregulated lncRNA SNHG4 was associated with NSCLC prognosis

In order to investigate the oncogenic and prognostic effect of lncRNA SNHG4 in NSCLC, we initially identified the expression of lncRNA SNHG4 in 50 cancerous tissues and matched noncancerous lung tissues via quantitative reverse transcription polymerase chain reaction (qRT-PCR). The results obtained demonstrated that lncRNA SNHG4 (Figure 1A) was upregulated in cancerous lung tissues relative to that of the normal adjacent tissues. Kaplan-Meier curves were constructed to illustrate the relationship between lncRNA SNHG4 and NSCLC patient prognosis. Patients with high lncRNA SNHG4 expression had shorter overall survival and disease-free survival when compared to those with low lncRNA SNHG4 expression (Figures 1B and 1C), highlighting the link between lncRNA SNHG4 and poor NSCLC prognosis. Next, we quantified the expression of lncRNA SNHG4 in human NSCLC cells (H1299, H1650, H1975, and SPCA1) and 16HBE cells by qRT-PCR. As we expected, the NSCLC cells exhibited higher expression of lncRNA SNHG4 when compared to the 16HBE cells ($p < 0.05$) (Figure 1D). Among them, lncRNA SNHG4 exhibited a relatively higher expression in the H1299 and H1975 cells, and thus H1299 and H1975 cells were selected for further experimentation. These results provided evidence indicating the negative association between lncRNA SNHG4 and the overall survival rate and disease-free survival rate of patients with NSCLC.

lncRNA SNHG4 regulated NSCLC cell proliferation, migration, invasion, and apoptosis by binding with miR-let-7e

After we identified that lncRNA SNHG4 is upregulated in NSCLC, we subsequently set out to elucidate the mechanism by which lncRNA SNHG4 contributes to NSCLC. Twenty-one common putative miRNAs binding with lncRNA SNHG4 were identified between two public databases, i.e., starBase and RNAInter, as depicted by the Venn diagram in Figure 2A. Among the 21 putative miRNAs, the miR-let-7 family had the highest score (0.7155) as per the RNAInter database. The starBase database indicated that miR-let-7e was downregulated in NSCLC tissues relative to that of the normal lung tissues (Figure 2B). Figure 2C depicts the miR-let-7e binding sites in the 3′ untranslated region (UTR) of lncRNA SNHG4 by starBase. In the following experiments, we successfully introduced miR-let-7e mimic and miR-let-7e inhibitor into H1299 cells, which was confirmed by qRT-PCR ($p < 0.05$) (Figure 2D). Luciferase activity at the promoter of the reporter gene containing the seed sequence in the 3′ UTR of lncRNA SNHG4 in lieu of the mutant reporter gene was reduced in the presence of miR-let-7e mimic (Figure 2E). An RNA pull-down assay revealed a strong binding relationship between lncRNA SNHG4 and miR-let-7e (Figure 2F). Likewise, we examined the expression of miR-let-7e in 50 cancerous tissues and matched noncancerous lung tissues by qRT-PCR and found that the expression of miR-let-7e was lower in cancerous tissues than that in noncancerous lung tissues (Figure 2G). Thus, we asserted the hypothesis that lncRNA SNHG4 influenced NSCLC cells by binding to miR-let-7e.

In order to verify this hypothesis, we constructed lncRNA SNHG4 knockdown H1299 and H1975 cells using lncRNA SNHG4-specific small interfering RNAs (siRNAs) and plasmids overexpressing lncRNA SNHG4. As indicated by qRT-PCR, the expression of lncRNA SNHG4 was markedly decreased while the expression of miR-let-7e was elevated in response to si-SNHG4-1 and si-SNHG4-2 treatment ($p < 0.05$), while si-SNHG4-1 exhibited a higher knockdown efficiency and was subsequently used in latter experiments (Figure 3A). A contrasting trend was found in the presence of overexpressed lncRNA SNHG4 ($p < 0.05$). The results of a Cell Counting Kit-8 (CCK-8) assay, monoclonal formation assays, a scratch test, and a Matrigel-based transwell invasion assay provided data suggesting that lncRNA SNHG4 knockdown arrested H1299 cell viability, colony formation, migration, and invasion (Figures 3B–3E). Annexin V/propidium iodide (PI)-labeled flow cytometric analysis revealed that lncRNA

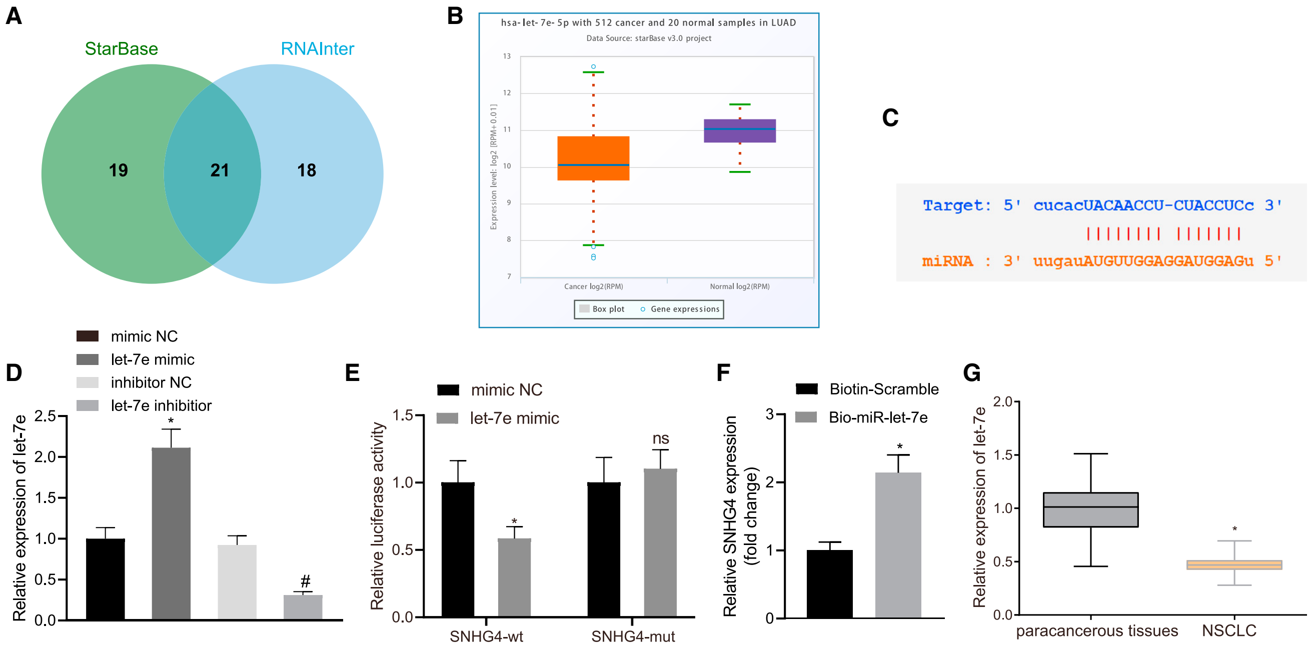

Figure 2. lncRNA SNHG4 binds with miR-let-7e

(A) Common 21 putative miRNAs binding with lncRNA SNHG4 stood out between two public databases, i.e., starBase (http://starbase.sysu.edu.cn/) and RNAInter (http://www.rna-society.org/rnainter/), shown by the Venn diagram. (B) Expression box of miR-let-7e between lung cancer tissues and normal tissues in the starBase database. (C) Putative miR-let-7e binding sites in the 3′ UTR of lncRNA SNHG4 by the starBase database. (D) The expression of miR-let-7e was determined by qRT-PCR in H1299 cells treated with miR-let-7e mimic and miR-let-7e inhibitor. (E) Luciferase activity at the promoter of the reporter gene containing the seed sequence in the 3′ UTR of lncRNA SNHG4 and, accordingly, the mutant reporter gene. (F) RNA pull-down of SNHG4 by miR-let-7e detected by qRT-PCR. (G) The expression of miR-let-7e was determined between NSCLC tissues (n = 50) and matched paracancerous lung tissues (n = 50) by qRT-PCR. In (D) and (E), *$p < 0.05$ (compared with H1299 cells treated with mimic NC) and $^{\#}p < 0.05$ (compared with H1299 cells treated with inhibitor NC) by unpaired t test. In (F), *$p < 0.05$ compared with biotin-scramble by an unpaired t test. In (G), *$p < 0.05$ compared with matched noncancerous lung tissues by a paired t test.

SNHG4 knockdown induced H1299 cell apoptosis (Figure 3F). The western blot analysis results indicated that lncRNA SNHG4 knockdown could elevate the expression of cleaved caspase-3 (Figure 3G). As expected, overexpressed (oe-)SNGH4 induced an opposite set of results in the H1299 cells. Hence, lncRNA SNHG4 knockdown H1299 cells were treated with miR-let-7e inhibitor. We observed that miR-let-7e inhibition enhanced H1299 cell viability, colony formation, migration, and invasion, reduced apoptosis, and diminished the expression of cleaved caspase-3 in lncRNA SNHG4 knockdown H1299 cells (Figures 3B–3G). Similar tendencies were observed in the H1975 cells (Figures S1A–S1G). Taken together, the aforementioned findings verified the regulatory role of lncRNA SNHG4 on proliferation, migration, and invasion, while indicating that the apoptosis of NSCLC cells was dependent on miR-let-7e.

lncRNA SNHG4 bound with miR-let-7e and upregulated KDM3A

Next, to identify the target gene of miR-let-7e in NSCLC, miRNA-mRNA prediction was performed using the RNA22, RNAInter, miRWalk, TargetScan, and microT databases, with two common target genes, i.e., PQLC2 and KDM3A, subsequently identified (Figure 4A). We then analyzed the differentially expressed genes between matching pairs of tumor-free lung and NSCLC specimens by using the Gene Expression Omnibus (GEO): GSE74706 dataset. The results revealed that the KDM3A gene exhibited a more distinct change between tumor-free lung and NSCLC tissue specimens than did the PQLC2 gene, and KDM3A was upregulated in NSCLC ($p < 0.05$) (Figure 4B). We subsequently evaluated the expression of KDM3A in 50 cancerous tissues and matched noncancerous lung tissues via qRT-PCR and immunohistochemical staining methods. The results demonstrated that KDM3A was upregulated in the cancerous lung tissues ($p < 0.05$) (Figures 4C and 4D). The expression of KDM3A was found to be positively correlated with the expression of lncRNA SNHG4 (Figure 4E). Figure 4F depicts the miR-let-7e binding sites in the 3′ UTR of lncRNA SNHG4 in accordance with starBase. Next, to confirm the notion that lncRNA SNHG4 functions as a ceRNA to regulate KDM3A, the luciferase activity at the promoter of the reporter gene containing the seed sequence in the 3′ UTR of KDM3A and the mutant reporter gene were determined, the results of which suggested that introduction of miR-let-7e mimic triggered a decrease in luciferase activity at the promoter of the reporter gene containing the seed sequence in the 3′ UTR of KDM3A compared with mutant reporter gene. We also observed that overexpression of lncRNA SNHG4 could restore the luciferase activity inhibited by the miR-let-7e mimic at the promoter of the reporter gene containing the seed sequence in the 3′ UTR of KDM3A (Figure 4G). Additionally, we performed anti-argonaute-2 (Ago2)-based RNA immunoprecipitation (RIP) assays in

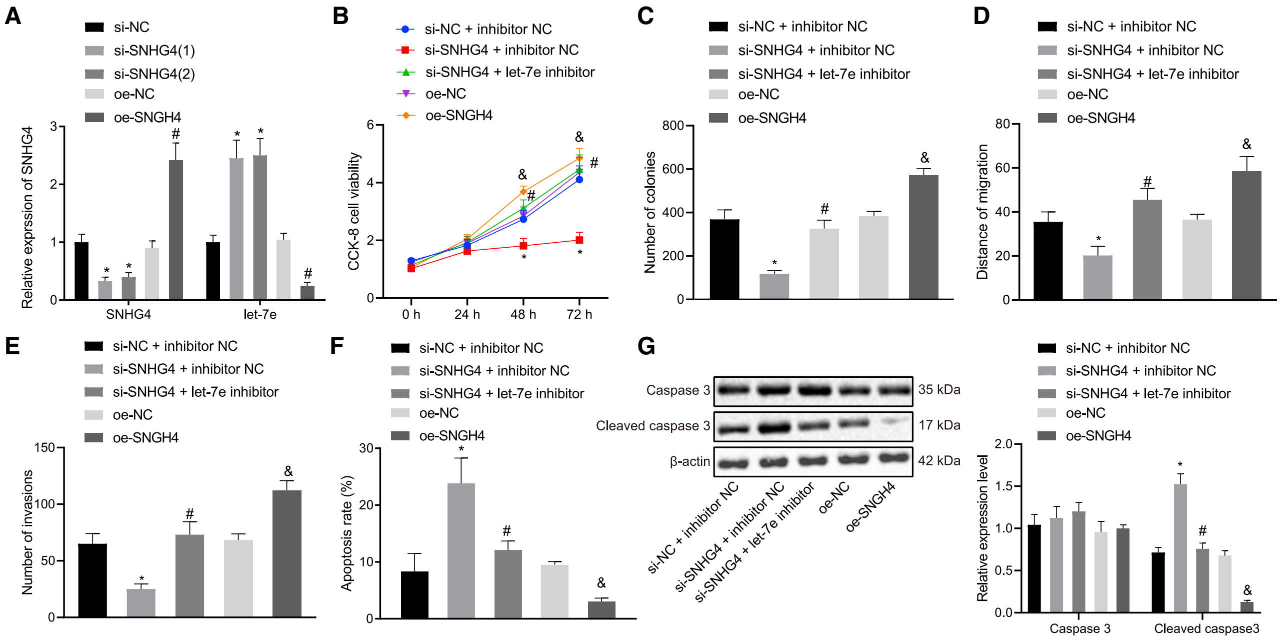

Figure 3. lncRNA SNHG4 mediates H1299 cell proliferation, migration, invasion, and apoptosis by binding with miR-let-7e

(A) Expression of lncRNA SNHG4 and miR-let-7e after knockdown of lncRNA SNHG4 in H1299 cells. (B) H1299 cell viability measured by CCK-8 assays. (C) Numbers of colonies derived from H1299 cells. (D) Scratch wound healing. (E) H1299 cells invading from Matrigel-coated upper transwell chambers into lower ones. (F) H1299 cell apoptosis determined by annexin V/PI-labeled flow cytometric analysis. (G) Western blots and quantification of caspase-3 and cleaved caspase-3 in H1299 cells, normalized to β-actin expression. $^{*}p < 0.05$ (compared with H1299 cells treated with scramble siRNA alone and/or inhibitor NC), $^{\#}p < 0.05$ (compared with H1299 cells treated with oe-NC alone or lncRNA SNHG4-specific siRNA plus inhibitor NC), and $^{\&}p < 0.05$ (compared with H1299 cells treated with oe-NC alone) by Tukey's test-corrected one-way ANOVA or Bonferroni-corrected repeated-measures ANOVA.

H1299 and H1975 cells transiently overexpressing lncRNA SNHG4. The results obtained demonstrated that endogenous KDM3A pull-down by Ago2 was significantly enriched in the lncRNA SNHG4-overexpressed H1299 and H1975 cells, suggesting that lncRNA SNHG4 and KDM3A shared a common miRNA ($p < 0.05$) (Figure 4H). The qRT-PCR and western blot analysis results indicated that miR-let-7e inhibited the expression of lncRNA SNHG4 and KDM3A ($p < 0.05$) (Figures 4I and 4J). The overexpression of lncRNA SNHG4 increased the expression of KDM3A in H1299 cells in the presence of miR-let-7e inhibitor. The results observed in the H1975 cells were similar to those in the H1299 cells (Figures S2A–S2C). Altogether, these results suggested that lncRNA SNHG4 functions as a ceRNA to regulate KDM3A by binding with miR-let-7e in NSCLC.

KDM3A functioned as an oncogene in NSCLC by inhibiting p21

The published literature has previously indicated that KDM3A, as a histone demethylase, inhibits the transcriptional activity of p53 by means of erasing p53-k372me1 in ovarian cancer.[19] Immunohistochemical staining provided evidence indicating that the expression of p53-k372me1 was significantly lower in the NSCLC tissues relative to the adjacent tissues ($p < 0.05$) (Figure 4D). In the subsequent experiments, we constructed KDM3A knockdown H1299 and H1975 cells by means of using KDM3A-specific siRNA. Western blot analysis results indicated that the expression of p53-k372me1 was enhanced following KDM3A knockdown ($p < 0.05$), while the expression of p53 did not differ ($p > 0.05$) (Figure 5A). Based on previous evidence, p53-k372me1 has been suggested to increase the transcriptional level of p21.[20] p21 was immunoprecipitated using p53 antibody relative to immunoglobulin G (IgG) by chromatin immunoprecipitation (ChIP) assays, and more p21 immunoprecipitated with p53 was examined following KDM3A knockdown ($p < 0.05$) (Figure 5B). Additionally, we also identified an increase in the expression of p21 and cleaved caspase-3 following KDM3A knockdown, which was negated in the event of knockdown of both KDM3A and p21 ($p < 0.05$) (Figure 5C). We subsequently set out to elucidate the role of KDM3A in NSCLC. The results of the CCK-8 assay, monoclonal formation assays, and annexin V/PI-labeled flow cytometric analysis revealed that KDM3A knockdown inhibited H1299 cell viability and colony formation, but it was able to induce apoptosis ($p < 0.05$) (Figures 5D–5F). The western blot analysis results demonstrated that KDM3A knockdown elevated the expression of cleaved caspase-3 (Figure 5C). In addition, the cell cycle was blocked in the G_1 phase by KDM3A knockdown, while the rates of apoptosis and cells at the G_1 phase were significantly reduced following suppression of p21 ($p < 0.05$) (Figure 5G). Next, KDM3A knockdown H1299 cells were treated with p21-specific siRNA. The knockdown of p21 enhanced cell viability, colony formation, migration, and invasion, reduced apoptosis, and diminished the expression of cleaved caspase-3 in KDM3A knockdown H1299 cells. Also, H1975 cells revealed similar

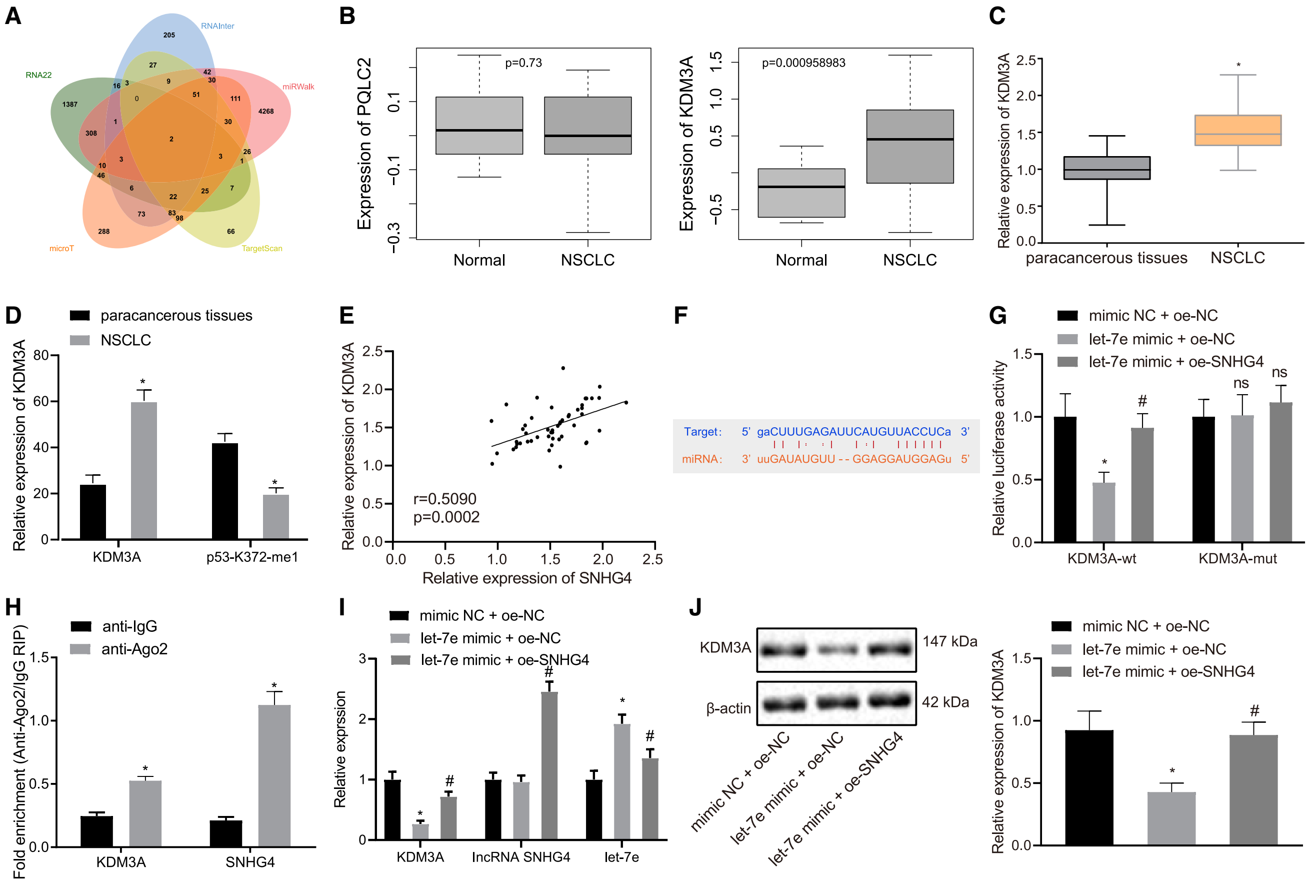

Figure 4. lncRNA SNHG4 bound with miR-let-7e and upregulated KDM3A

(A) Putative target genes of miR-let-7e among the RNA22 (https://cm.jefferson.edu/rna22/), RNAInter (http://www.rna-society.org/rnainter/), miRWalk (http://mirwalk.umm.uni-heidelberg.de/), TargetScan (http://www.targetscan.org/vert_72/), and microT (http://diana.imis.athena-innovation.gr/DianaTools/index.php?r=microT_CDS/index) databases. (B) Expression box of PQLC2 and KDM3A between lung cancer tissues and normal tissues in the starBase database. (C) The expression of KDM3A was determined by qRT-PCR in cancerous tissues (n = 50) and matched noncancerous lung tissues (n = 50). (D) Immunohistochemical staining for KDM3A and p53-k372me1 in cancerous tissues (n = 50) and matched noncancerous lung tissues (n = 50). (E) Pearson correlation analysis of KDM3A and lncRNA SNHG4. (F) Putative miR-let-7e binding sites in the 3′ UTR of KDM3A by the starBase database. (G) Luciferase activity at the promoter of the reporter gene containing the seed sequence in the 3′ UTR of KDM3A and, accordingly, the mutant reporter gene in the presence of miR-let-7e mimic and/or expression vector containing the lncRNA SNHG4. (H) Anti-Ago2 RIP in H1299 cells transiently overexpressing lncRNA SNHG4. (I) The expression of KDM3A was determined by qRT-PCR in H1299 cells. (J) Western blots and quantification of KDM3A in H1299 cells, normalized to β-actin expression. *$p < 0.05$ (compared with H1299 cells treated with oe-NC with or without mimic NC) and $^{\#}p < 0.05$ (compared with H1299 cells treated with miR-let-7e mimic with oe-NC) by unpaired t test or Tukey's test-corrected one-way ANOVA. In (C) and (D), *$p < 0.05$ compared with matched noncancerous lung tissues by paired t test.

results (Figures S3A–S3G). The aforementioned findings revealed that KDM3A promoted the proliferation of NSCLC cells by inhibiting p21.

lncRNA SNHG4 promoted the tumorigenicity of human NSCLC cells *in vivo*

Finally, we set out to evaluate the effects of lncRNA SNHG4 on the tumorigenicity of human NSCLC cells. H1299 cells were treated with SNHG4-specific short hairpin RNA (shRNA) alone or in combination with p21-specific shRNA or p21-specific shRNA alone. qRT-PCR provided data suggesting that lncRNA SNHG4 knockdown diminished lncRNA SNHG4 expression in the H1299 cells, while no significant difference was detected in relationship to the expression of lncRNA SNHG4 when p21 was knocked down in the presence of sh-lncRNA SNHG4 ($p < 0.05$). lncRNA SNHG4 knockdown enhanced the expression of p21 ($p < 0.05$), a finding of which was inhibited following the addition of p21-specific siRNA in H1299 cells (Figure 6A). Western blot analysis revealed that lncRNA SNHG4 knockdown could reduce the expression of KDM3A, but it increased the expression of p21 and cleaved caspase-3 in H1299 cells ($p < 0.05$) (Figure 6B). Also, KDM3A expression did not differ significantly in the presence of sh-p21 alone, while the expression of p21 and cleaved caspase-3 was significantly decreased in H1299 cells ($p < 0.05$). Following both lncRNA SNHG4 and p21 knockdown, the expression of KDM3A failed to exhibit a significant difference, while cleaved caspase-3 was reduced when compared with treatment with sh-SNHG4 alone in H1299 cells.

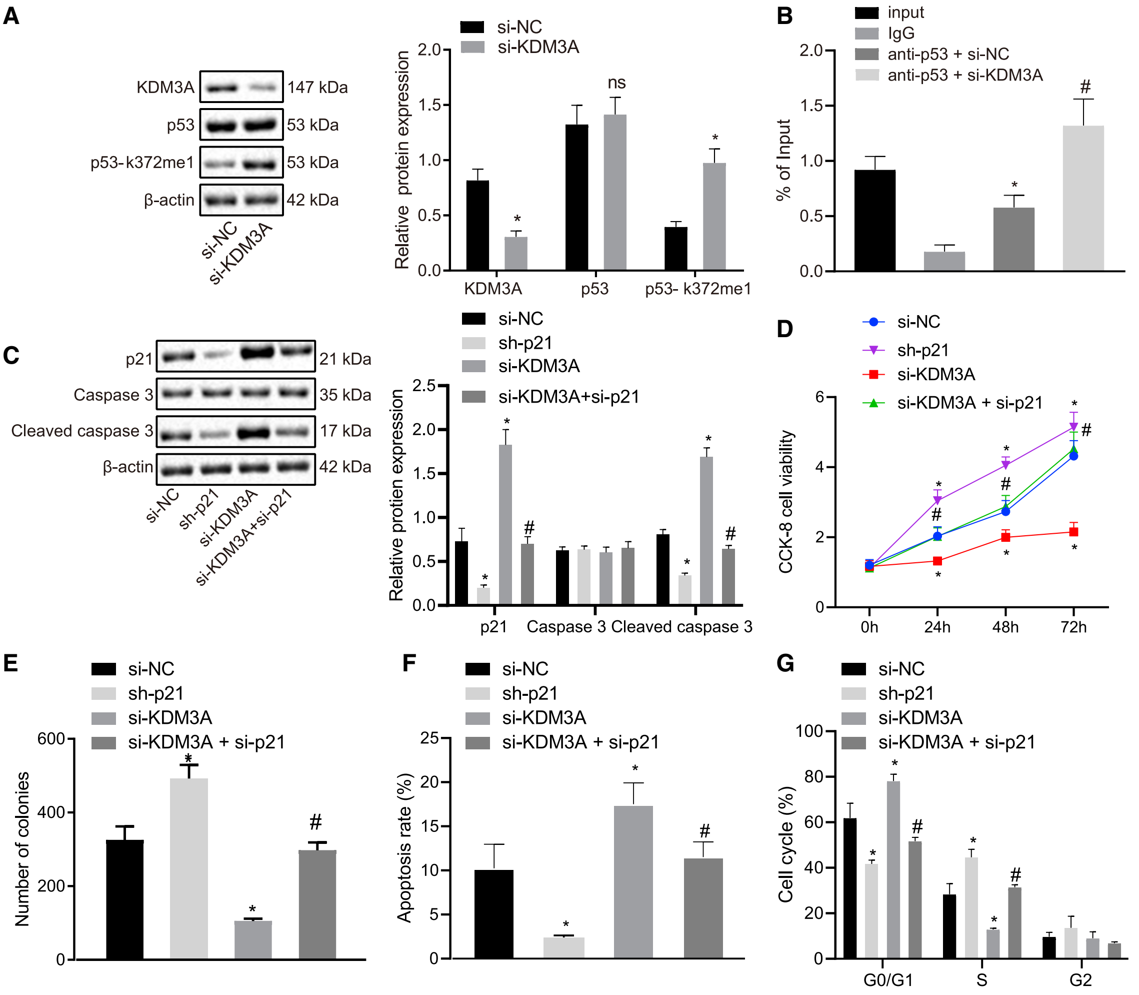

Figure 5. KDM3A functioned as an oncogene in NSCLC by inhibiting p21

(A) Western blots and quantification of KDM3A, p53-k372me1, and p53 in H1299 cells, normalized to β-actin expression. (B) p21 was immunoprecipitated using p53 antibody relative to IgG by ChIP assays. (C) Western blots and quantification of p21, cleaved caspase-3, and caspase-3 in H1299 cells, normalized to β-actin expression. (D) H1299 cell viability was measured by CCK-8 assays. (E) Numbers of colonies derived from H1299 cells. (F) H1299 cell apoptosis determined by annexin V/PI-labeled flow cytometric analysis. (G) H1299 cell cycle determined by flow cytometric analysis. *$p < 0.05$ (compared with scramble siRNA or IgG antibody) and $^{\#}p < 0.05$ (compared with si-KDM3A or anti-p53 with si-NC) by unpaired t test, Tukey's test-corrected one-way ANOVA, or Bonferroni-corrected repeated-measures ANOVA.

Next, BALB/c nude mice were subcutaneously injected with H1299 cells stably expressing SNHG4-specific shRNA and/or p21-specific shRNA. We also found that 30 days later, lncRNA SNHG4 knockdown led to reductions in the weight and volume of the subcutaneous xenotransplanted tumors of the human H1299 cells, while sh-p21 alone triggered an opposite trend in results. Moreover, additional sh-p21 treatment counterweighed the action of sh-SNHG4 on tumor weight and volume (Figures 6C and 6D). Immunohistochemical staining was performed on the mouse tumor tissues in order to identify Ki67-positive cells and measure cell proliferation. A reduction in the Ki67-positive cells was detected in the presence of sh-SNHG4 alone, while elevated Ki67-positive cells were noted in the presence of sh-p21 alone. The number of Ki67-positive cells was increased by sh-p21 when SNHG4 was knocked down (Figure 6E). Terminal deoxynucleotidyl transferase-mediated 2′-deoxyuridine 5′-triphosphate nick end labeling (TUNEL) staining revealed that cell apoptosis was significantly promoted by sh-SNHG4 while it was suppressed by sh-p21. Furthermore, sh-p21 was observed to be capable of overriding the action of sh-SNHG4 on cell apoptosis (Figure 6F). Furthermore, expression of KDM3A, p21, cleaved caspase-3, and caspase-3 biochemically from tumor samples changed similarly as observed in H1299 cells (Figures 6G and 6H). Taken together, the results obtained suggested that lncRNA SNHG4 enhances the expression of KDM3A and p21, which ultimately promotes the tumorigenicity of human NSCLC cells *in vivo*.

DISCUSSION

NSCLC represents a well-documented cause of cancer-related mortality on a global scale.[2] In recent years, significant forward

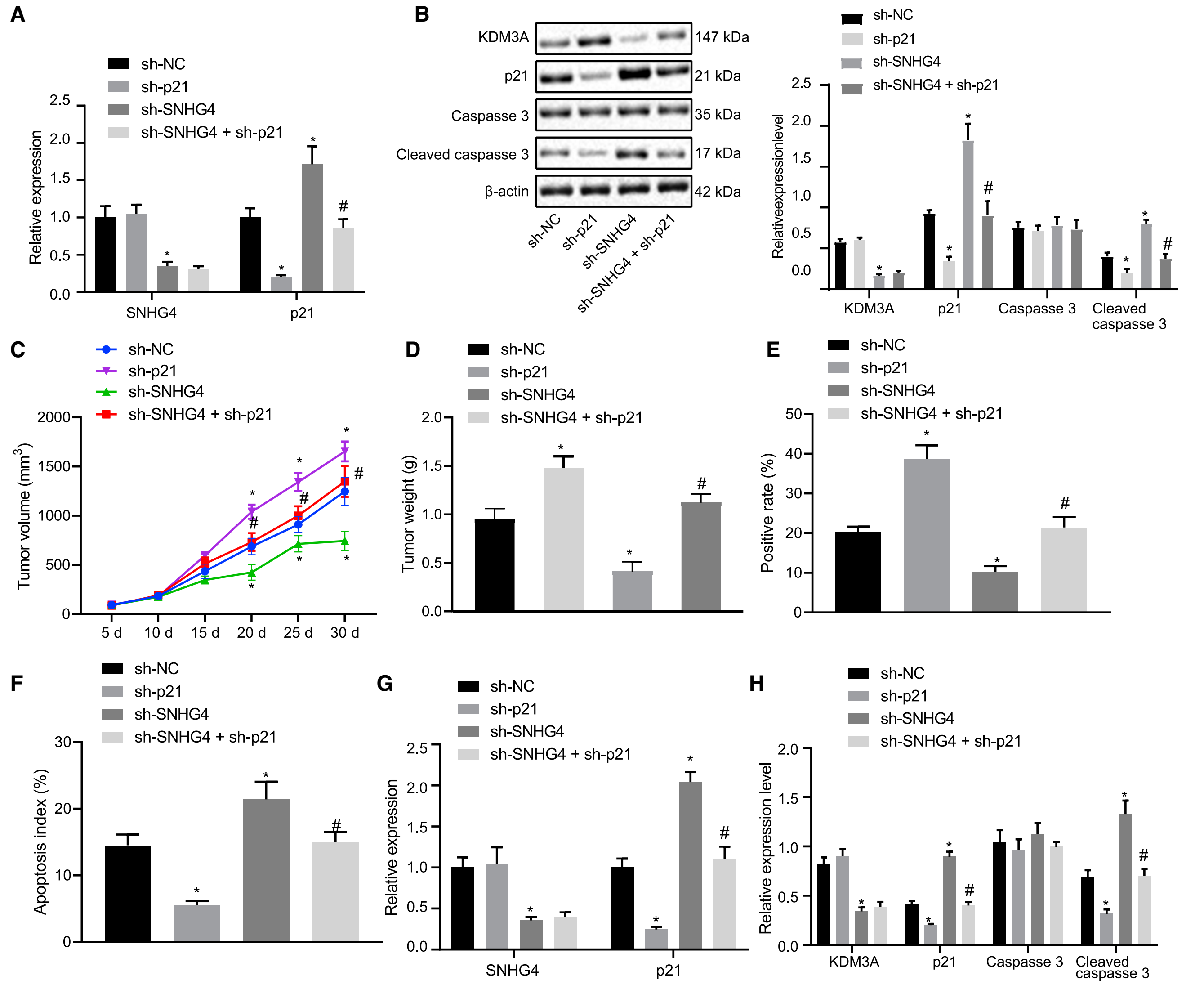

Figure 6. lncRNA SNHG4 promoted the tumorigenicity of human NSCLC cells *in vivo* by increasing KDM3A and p21 expression

(A) Verification of lncRNA SNHG4 and/or p21 knockdown by qRT-PCR. (B) Western blots and quantification of KDM3A, p21, cleaved caspase-3, and caspase-3 in H1299 cells, normalized to β-actin expression. (C) The growth of mouse xenotransplanted tumors of H1299 cells at indicated time points. (D) Weight of mouse xenotransplanted tumors of H1299 cells. (E) Ki67-positive cells detected by immunohistochemical staining. (F) Cell apoptosis detected by TUNEL staining. (G) lncRNA SNHG4 and p21 expression detected by qRT-PCR. (H) Western blot analysis of KDM3A, p21, cleaved caspase-3, and caspase-3 in tumor tissues, normalized to β-actin expression. *$p < 0.05$ (compared with mice treated with scramble shRNA) and $^{\#}p < 0.05$ (compared with mice treated with lncRNA SNHG4-specific shRNA) by Tukey's test-corrected one-way ANOVA or Bonferroni-corrected repeated-measures ANOVA.

strides have been made in the treatment of NSCLC; however, the overall survival rate for NSCLC remains poor.[21] Accumulating evidence continues to implicate lncRNAs in various NSCLC processes, such as cell proliferation, migration, invasion, and apoptosis.[22] Notably, lncRNA SNHG4 has been reported to promote the metastasis of lung cancer cells,[12] while the impact of lncRNA SNHG4 on NSCLC and its downstream mechanism remains largely unclear. Key observations made during the present study revealed that SNHG4 sponging miR-let-7e promoted cell migration and invasion while suppressing the apoptosis of NSCLC cells and consequently facilitated the progression of NSCLC by regulating KDM3A.

The dysregulated expression of lncRNAs has been previously reported to play a notable role in the development of NSCLC.[9] For example, the aberrant expression of lncRNA HOXA-AS3 expression was previously shown to influence the proliferation, differentiation, invasion, and metastasis of NSCLC cells.[8] lncRNA SNHG1 had been reported to be highly expressed in NSCLC, while the overexpression of lncRNA SNHG1 has been reported to enhance tumor cell

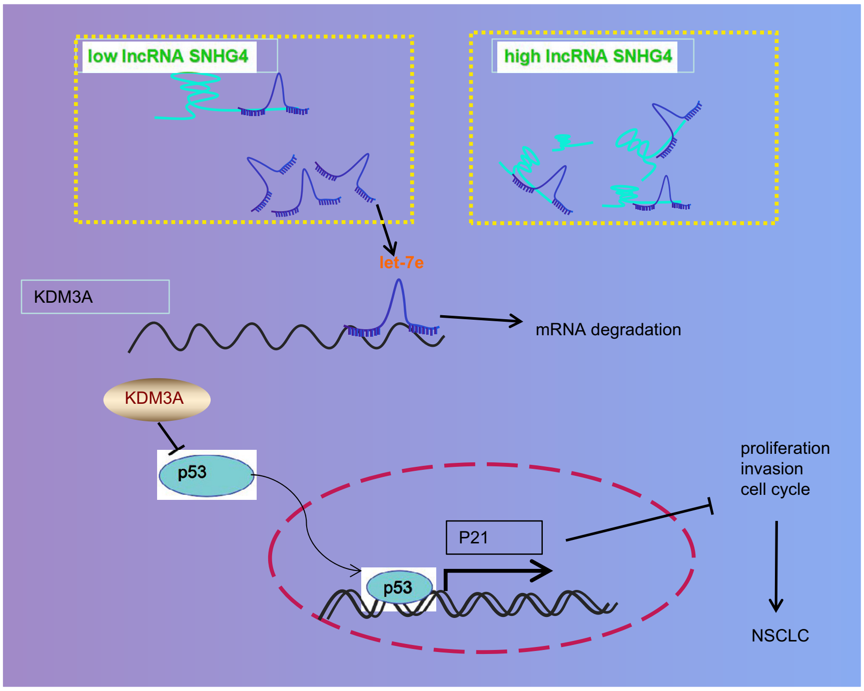

Figure 7. Systematic diagram showing how the lncRNA SNHG4/let-7e/KDM3A/p21 pathway is involved in the development of NSCLC

lncRNA SNHG4 binds with miR-let-7e and thus positively regulates the expression of KDM3A. KDM3A, a histone demethylase, inhibits the transcriptional activity of p53 by erasing p53-k372me1 and reduces the expression of p21. In this context, reduced p21 promotes the development of NSCLC.

metastasis and further aggravate NSCLC.[23] As one of the members of the SNHGs family, lncRNA SNHG4 continues to attract significant attention with regard to its role and expression in numerous human tumors.[24] Elevated lncRNA SNHG4 expression has been detected in prostate cancer, while depleted lncRNA SNHG4 has been demonstrated to suppress prostate cancer cell growth, migration, and invasion.[11] Similarly, lncRNA SNHG4 has been reported to be upregulated in lung cancer cells, while the silencing of lncRNA SNHG4 plays a contributory role in the inhibition of lung cancer progression *in vitro* and *in vivo*.[12] Our results illustrated that lncRNA SNHG4 was upregulated in both NSCLC tissues and cells. Moreover, silencing of lncRNA SNHG4 was able to inhibit the migration and invasion and promote apoptosis of NSCLC cells, ultimately suppressing the initiation and progression of NSCLC both *in vitro* and *in vivo*. These findings lend crucial support to the notion of lncRNA SNHG4 knockdown as a potential therapeutic target to delay the process of NSCLC.

In addition, our observations indicated that lncRNA SNHG4 could facilitate the progression of NSCLC by acting as a ceRNA of miR-let-7e. Emerging evidence has suggested that lncRNAs may regulate the expression of target genes by binding to miRNAs, which could serve as novel therapeutic approach for NSCLC treatment.[25,26] Moreover, our results indicated that miR-let-7e was poorly expressed in NSCLC tissues, while the inhibition of miR-let-7e leads to an increase in cell migration and invasion and suppression of apoptosis in NSCLC. Additionally, miR-let-7e had been demonstrated to be downregulated in NSCLC,[16] with Zhang et al.[27] asserting that miR-let-7e may function as a potential biomarker and target for diagnosis and prognosis of NSCLC. In addition, a recent study suggested that lncRNA SNHG4 regulates the development of lung cancer via miR-98-5p.[12] Another study provided evidence suggesting that lncRNA NEAT1 could competitively bind to miR-let-7a to regulate both NSCLC cell proliferation and metastasis.[28] The aforementioned reports and results suggest that the etiology and development of NSCLC may be regulated by multiple lncRNAs and miRNAs, which requires further exploration in future research.

Both the bioinformatics analysis and related experimental results provided data suggesting that lncRNA SNHG4 could target KDM3A by binding to miR-let-7e. KDM3A was a lysine demethylase for the non-histone protein p53, playing an inhibitory role in tumor growth of different cancers,[19,29] including lung cancer.[18] Moreover, Wade et al.[30] illustrated that knockdown of KDM3A inhibited the proliferation of tumor cells and was linked to drug resistance. Similarly, in the current study, our results also supported the notion that KDM3A overexpression enhances cell viability and impedes apoptosis by inhibiting the p21 pathway. Interestingly, many studies have uncovered that the effects of several lncRNAs on NSCLC are associated with p21 signaling.[31–33] Furthermore, as a crucial sensor of anti-proliferative signals, p21 exhibits paradoxical tumor-promoting activities in different cancers,[34] implying that lncRNA SNHG4 might play a role in other cancers by mediating the p21 pathway.

In conclusion, the key findings of our study demonstrate that lncRNA SNHG4 is highly expressed in NSCLC and regulates KDM3A by means of competitively binding to miR-let-7e (Figure 7). Additionally, silencing lncRNA SNHG4 or KDM3A was found to restrict the viability and metastasis of NSCLC cells, lengthen the G_1 phase of the cell cycle, and increase apoptosis. KDM3A was found to impede the methylation of p53K372, which subsequently restricted the activity of p53 and the expression of p21. The central findings of our study provide an interesting basis for potential targets for NSCLC prevention and treatment.

MATERIALS AND METHODS

Ethics statement

All human subjects included in our study provided signed informed consent documentation prior to enrollment as per the guidelines of the Ethics Committee of Linyi People's Hospital. All animal

Table 1. Primer sequences used for qRT-PCR

Target	Forward (5′→3′)	Reverse (5′→3′)
U6	AATTGGAACGATACAGAGAAGATTAGC	TATGGAACGCTTCACGAATTTG
GAPDH	GAGAGACCCCACTTGCTGCCA	GGAAGAAGTTCCCATCGTCA
SNHG4	GGCTAGAGTACAGTGGCTCG	GCAAATCGCAAGGTCAGG
KDM3A	ATGCCCACACAGATCATTCC	CTGCACCAAGAGTCGGTTTT
miR-let-7e	GGTGAGGTAGTAGGTTGTATGG	TGTCGTGGAGTCGGCAATTG

qRT-PCR, quantitative reverse transcriptase polymerase chain reaction; U6, hypothetical protein; GAPDH, glyceraldehyde-3-phosphate dehydrogenase; SNHG4, small nucleolar RNA host gene 4; KDM3A, lysine demethylase 3A; miR-let-7e, microRNA-let-7e.

experiments were performed with the approval of the Animal Use and Care Committee of Linyi People's Hospital with the procedures conducted in strict accordance with the *Guide for the Care and Use of Laboratory Animals*.

Bioinformatics analysis

The NSCLC-related microarray GEO: GSE74706 dataset (https://www.ncbi.nlm.nih.gov/geo/) was obtained, comprised of 18 normal samples and 18 NSCLC samples, after which a differential analysis was performed using the limma package of R language with |log fold change| >1 and a p value <0.05 as the threshold values. The downstream miRNAs of lncRNA SNHG4 were predicted through starBase (http://starbase.sysu.edu.cn/) and RNAInter (http://www.rna-society.org/rnainter/). An interaction was obtained using jvenn (http://jvenn.toulouse.inra.fr/app/example.html) for further screening. The miRNA target genes were predicted using RNA22 (https://cm.jefferson.edu/rna22/), RNAInter, miRWalk (http://mirwalk.umm.uni-heidelberg.de/), TargetScan (http://www.targetscan.org/vert_72/), and microT (http://diana.imis.athena-innovation.gr/DianaTools/index.php?r=microT_CDS/index), followed by intersection through jvenn.

Human tissue specimens and follow-up

Both tumor and adjacent non-tumor tissue samples were collected from 50 patients (33 males and 17 females) who were previously diagnosed with NSCLC based on histopathological examination performed at the Linyi People's Hospital between January 2009 and January 2014. The age of the 86 patients ranged from 45 to 73 years (an average age of 59.78 ± 7.85 years), with the tumor size ranging from 0.59 to 4.50 cm (an average size of 2.62 ± 0.94 cm). There were 11 cases confirmed to be at stage I of tumor node metastasis, 19 stage II cases, and 20 stage III cases. All patients had not received any radiotherapy or chemotherapy prior to surgery. All patients were followed up for a period of 60 months until January 2019. The follow-up period ranged from 18 to 60 months (average, 51.24 months).

Cell lines and cell transfections

Four types of human lung cancer cell lines were selected, namely H1299, H1650, H1975, and SPCA1, in addition to normal human bronchial epithelial cell line 16HBE. H1299, H1975, H1650, and HEK293T cells were purchased from the ATCC (Manassas, VA, USA), and H1975, SPCA1, and 16HBE cells were from the cell bank of the Chinese Academy of Sciences. The miR-let-7e mimic, inhibitor, mimic negative control (NC), and inhibitor NC were purchased from GenePharma (Shanghai, P.R. China). The cells were seeded into 12-well plates at a density of 3 × 10^5 cells/well. Upon reaching 80% confluence, Lipofectamine 2000 (Invitrogen, Thermo Fisher Scientific, Waltham, MA, USA) was used to conduct transfection at a final concentration of 50 nM. Serum-free Opti-MEM (minimal essential medium) (Gibco, Grand Island, NY, USA) (250 μL) was respectively diluted with the target plasmid (4 μg) and Lipofectamine 2000 (10 μL), with the solutions subsequently mixed in a gentle manner. After standing at room temperature for 5 min, the above two solutions were mixed. After 20 min, the mixture was added to the cells and incubated at 37°C with 5% CO_2 for 6 h. The culture medium was subsequently renewed with a complete medium. The cells were cultured in a continuous manner for 48 h and finally collected. Next, shRNAs were designed and inserted into the pGPH1/Neo (GenePharma) by Invitrogen, with 75 pmol of them delivered into cells using Lipofectamine 3000 (Invitrogen).

qRT-PCR

The total RNA of the tissues and cells was extracted using TRIzol (Invitrogen). cDNA was generated using the Mir-X miRNA first-strand synthesis kit for miRNA as well as a commercially available kit (RR047A) for mRNA, with both used in strict accordance with the instructions provided by the manufacturer (Takara, Dalian, P.R. China). Quantification of miR-let-7e expression was evaluated using a Mir-X miRNA qRT-PCR TB Green kit (Takara). Quantification of lncRNA SNHG4 and KDM3A mRNA expression was performed with the SYBR Premix Ex Taq II (perfect real time) kit (DRR081, Takara) using a ABI Prism 7300 system (Applied Biosystems, Foster City, CA, USA). The expression of miR-let-7e was normalized to the expression of U6, and the expression of lncRNA SNHG4 and KDM3A was normalized to the expression of glyceraldehyde-3-phosphate dehydrogenase (GAPDH). The results were calculated using the $2^{-\Delta\Delta CT}$ method. The primer sequences are depicted in Table 1.

CCK-8 assay

Cell viability was examined via a CCK-8 assay. Briefly, the cells were inoculated into a 96-well plate with 2 × 10^3 cells/mL and 100 μL/well and incubated with 10 μL of CCK-8 solution (Dojindo Laboratories, Kumamoto, Japan) for 4 h. Two hours later, the media were renewed using 150 μL of dimethyl sulfoxide (Sigma-Aldrich, St. Louis, MO,

USA) in each well to dissolve the formazan crystals. Absorbance was read at 450 nm using a microplate reader (Thermo Scientific, Helsinki, Finland) at 24, 48, 72, and 96 h after inoculation, with growth curves plotted accordingly. The results were recorded based on the findings of three separate assays.

Monoclonal formation assay

The transfected cells were seeded into six-well plates (2×10^3 cells/well) and maintained in the Dulbecco's modified Eagle's medium (DMEM) containing 10% fetal bovine serum (FBS). Two weeks later, the cells were fixed using 95% methanol for 20 min and stained with 0.1% crystal violet (JissKang, Qingdao, Shandong, P.R. China). The colonies were counted under a microscope (37XF-PC, Shanghai Optical Instrument Factory, Yangpu, Shanghai, P.R. China).

Scratch test

The cells were seeded into a six-well plate with 1×10^5 cells/well. A thin scratch was created along the center of each well using a sterile 200-μL pipette tip (the width of each scratch was the same). Next, in an attempt to evaluate wound closure, six fields were selected, with the cells imaged at 0 and 24 h after incubation in serum-free medium with 1 μg/mL mitomycin. The cells in the wound area were counted and analyzed using counting software.

Matrigel-based transwell invasion assay

Cell invasion assays were performed using transwell chamber assays (Corning Life Sciences, Corning, NY, USA) as per the instructions provided by the manufacturer. Briefly, the cells were resuspended into 1×10^5 cells/mL using serum-free Roswell Park Memorial Institute 1640 medium and placed into the upper chambers coated with 20 μL of Matrigel (BD Biosciences, Bedford, MD, USA) that had been previously diluted with serum-free DMEM. After a 24-h period of incubation at 37°C, the cells that had been transferred to the lower chamber containing 10% FBS (Gibco, Grand Island, NY, USA)-supplemented DMEM (600 μL) were stained with 0.1% crystal violet followed by counting in six random fields per well using an Olympus inverted microscope (37XF-PC, Olympus, P.R. China).

Flow cytometric analysis

The cell nuclei were stained with PI using a kit (FXP031-100, Beijing 4A Biotech, P.R. China) and analyzed using a flow cytometer (FACScan; BD Biosciences) equipped with CellQuest software (BD Biosciences). PI was used in conjunction with annexin V (FXP018-100 kit, Beijing 4A Biotech, P.R. China) in order to determine whether cells were viable, apoptotic, or necrotic based on flow cytometry.

Cell cycle analysis

Cells exhibiting logarithmic growth were plated in a six-well plate at a density of 2×10^5 cells/well. Upon reaching 90% confluence, the cells were washed twice with phosphate-buffered saline and fixed with precooled ethanol at 4°C overnight. Next, the cells were incubated with 500 μL of PI/RNase A staining buffer (PI/RNase A at 9:1) under conditions void of light for 30 min and subsequently detected using a flow cytometer.

Western blot analysis and antibodies

The cells were lysed using protease inhibitor-contained radioimmunoprecipitation assay buffer (R0010, Solarbio, P.R. China) for protein extraction. After sodium dodecyl sulfate polyacrylamide gel electrophoresis analysis, the protein was subsequently transferred onto polyvinylidene fluoride membranes and probed with the following primary antibodies (Abcam, Cambridge, UK): mouse anti-KDM3A (1:1,000, ab91252), mouse anti-phosphorylated (p-)53 (1:1,000, ab1101), rabbit anti-cleaved caspase-3 (1:500, ab49822), rabbit anti-p53-k372me (1:10,000, ab16033), rabbit anti-caspase-3 (1:2,000, ab13847), and mouse anti-β-actin (1:10,000, ab8226). Western blots were exposed to horseradish peroxidase-coupled goat anti-rabbit IgG (ab205718, 1:20,000) or goat anti-mouse IgG (ab205719, 1:20,000) and enhanced chemiluminescence detection reagents (BB-3501, Amersham Pharmacia Biotech, Little Chalfont, UK). Target protein bands were quantified using Quantity One v4.6.2 software, with β-actin used for normalization.

Dual-luciferase reporter gene assay

The untranslated region at the 3′ UTR of KDM3A wild-type (KDM3A-WT) or SNHG4-WT containing the putative miR-let-7e binding sites, KDM3A mutated in the putative miR-let-7e binding sites (SEMA6B-MUT) or SNHG4-WT was inserted into the PYr-Mir-Target luciferase vectors, respectively. The desired luciferase vectors with either miR-let-7e mimic or mimic NC were co-transfected into the HEK293T cells using Lipofectamine 2000 reagent (Invitrogen). After 24 h of transfection, the cells were harvested and lysed accordingly. Renilla luciferase working solution (100 μL) and firefly luciferase working solution (100 μL) were mixed separately with the cell lysate (100 μL) supernatant. The multifunctional microplate reader SpectraMax M5 (Molecular Devices, Shanghai, P.R. China), at an interval time of 2 s and a measurement time of 10 s, was used to determine the Renilla luciferase and firefly luciferase activities.

RNA pull-down assay

Next, total RNA was extracted from NSCLC cells, after which 500 μg of streptavidin magnetic beads was permitted to bind to 200 pmol of biotin-labeled miR-let-7e mimic, followed by incubation with RNA at room temperature for 30 min. After elution, the pulled-down RNA complex was collected, after which the expression of lncRNA SNHG4 was determined by qRT-PCR.

RIP assay

H1299 cells treated with either the expression vector containing the lncRNA SNHG4 or empty vector were subject to RIP assays using a Magna RIP kit (17-701, Millipore, Billerica, MA, USA) and anti-Ago antibody (ab32381, 1:100, Abcam) in accordance with the manufacturer's instructions. Immunoprecipitated RNA and total RNA from the whole-cell lysates (input controls) were extracted for real-time qPCR analysis.

ChIP assay

Enrichment of p53 in the p21 promoter region was evaluated by ChIP assays (Upstate Biotechnology, Lake Placid, NY, USA). Briefly, the

cells (2×10^6) were maintained with 5 mmol dimethyl 3,3′- dithiobispropionimidate-HCl (Pierce Biotechnology, Waltham, MA, USA) for 30 min and subsequently fixed with formaldehyde to generate DNA-protein cross-links. The enriched DNA samples were analyzed by qRT-PCR using 5′-GTGGCTCTGATTGGCTTTCTG-3′ (forward primer of p21) and 5′-CTGAAAACAGGCAGCCCAAG-3′ (reverse primer of p21).

Tumorigenicity assays of human NSCLC cells *in vivo*

Eighteen specific pathogen-free-conditioned female BALB/c nude mice (aged 3–6 weeks, weighing 20–25 g, Hunan SJA Laboratory Animal, P.R. China) were subcutaneously injected with H1299 cells treated with SNHG4-specific shRNA or p21-specific shRNA alone or in combination with p21-specific shRNA. Tumor growth was evaluated every 5 days during a period of 6 weeks. All mice were euthanized by means of cervical dislocation. Fresh tumor tissues were fixed and paraffin-embedded. Finally, the expression levels of KDM3A, p21, caspase-3, and SNHG4 in the tumor tissues were determined.

Immunohistochemistry

The tumor tissues and adjacent tissues from patients or tumor tissues from nude mice were paraffin-embedded, cut into 5-μm sections, and made into slides. The slides were then subjected to immunohistochemical staining using mouse antibodies (Abcam) to KDM3A (ab91252, 1:200), p53-k372me1 (ab1101, 1:1,000), and anti-Ki67 (ab16667, 1:1,000). Visualization was performed using 3,3′-diaminobenzidine (DAB) (DA1010, Solarbio, Beijing, P.R. China). Five microscopic views were randomly captured to evaluate staining intensity.

TUNEL staining

The paraffin-embedded tumor tissue slices were baked at 50°C for 2 h, dewaxed by xylene, and dehydrated with 100%, 95%, 90%, 80%, and 70% gradient alcohol. The slices were permitted to react with 3% H_2O_2 at room temperature with Proteinase K at 37°C under conditions void of light for 10 min, followed by a 2-h period of incubation with labeling buffer (20 μL/slice) in a wet box. Each slice underwent reaction at room temperature following the addition of 50 μL of blocking solution for 30 min. Fifty μL of biotinylated anti-digoxin was added for 30-min incubation in a wet box at 37°C. Every 50 μL of streptavidin-biotin complex was then added to each slice and allowed to react for 30 min in a wet box at 37°C. The slices were developed with DAB for 6 min, counterstained with hematoxylin, dehydrated with gradient alcohol, cleared with xylene, mounted with resin, and microscopically observed (Olympus CX41). The apoptotic nuclei were tan-stained while the normal nuclei were stained blue. In total, 500 cells were randomly counted from each slice in order to calculate the percentage of apoptotic cells.

Statistical analysis

Measurement data are expressed as the mean ± standard deviation based on the findings of at least three independent experiments performed in triplicate. Statistical comparisons were performed using a Student t test when conducting comparisons between two groups or by Tukey's test-corrected one-way analysis of variance (ANOVA) when more than two groups were compared. Variables were analyzed at different time points using Bonferroni-corrected repeated-measures ANOVA. The Pearson correlation coefficient was applied for statistical correlation. Survival curves were plotted using Kaplan-Meier's method and analyzed using a log-rank test. All statistical analyses were performed using SPSS 21.0 software (IBM, Armonk, NY, USA), with two-tailed $p < 0.05$ deemed to be indicative of statistical significance.

SUPPLEMENTAL INFORMATION

Supplemental Information can be found online at https://doi.org/10.1016/j.omto.2020.12.010.

ACKNOWLEDGMENTS

We acknowledge and appreciate our colleagues for their valuable efforts and comments on this paper.

AUTHOR CONTRIBUTIONS

F.W. and Q.Q. conceived and designed research; F.W. analyzed data, prepared figures, and drafted the manuscript; Q.Q. performed experiments, interpreted results of experiments, and edited and revised the manuscript. Both authors approved the final version of the manuscript.

DECLARATION OF INTERESTS

The authors declare no competing interests.

REFERENCES

1. Tan, W.L., Jain, A., Takano, A., Newell, E.W., Iyer, N.G., Lim, W.T., Tan, E.H., Zhai, W., Hillmer, A.M., Tam, W.L., and Tan, D.S.W. (2016). Novel therapeutic targets on the horizon for lung cancer. Lancet Oncol. *17*, e347–e362.
2. Tan, S., Gou, Q., Pu, W., Guo, C., Yang, Y., Wu, K., Liu, Y., Liu, L., Wei, Y.Q., and Peng, Y. (2018). Circular RNA F-circEA produced from *EML4-ALK* fusion gene as a novel liquid biopsy biomarker for non-small cell lung cancer. Cell Res. *28*, 693–695.
3. Zhang, Y., Chen, W.J., Gan, T.Q., Zhang, X.L., Xie, Z.C., Ye, Z.H., Deng, Y., Wang, Z.F., Cai, K.T., Li, S.K., et al. (2017). Clinical significance and effect of lncRNA HOXA11-AS in NSCLC: a study based on bioinformatics, in vitro and in vivo verification. Sci. Rep. *7*, 5567.
4. Miller, K.D., Siegel, R.L., Lin, C.C., Mariotto, A.B., Kramer, J.L., Rowland, J.H., Stein, K.D., Alteri, R., and Jemal, A. (2016). Cancer treatment and survivorship statistics, 2016. CA Cancer J. Clin. *66*, 271–289.
5. Bao, S.M., Hu, Q.H., Yang, W.T., Wang, Y., Tong, Y.P., and Bao, W.D. (2019). Targeting epidermal growth factor receptor in non-small-cell-lung cancer: current state and future perspective. Anticancer. Agents Med. Chem. *19*, 984–991.
6. Baskar, R., Dai, J., Wenlong, N., Yeo, R., and Yeoh, K.W. (2014). Biological response of cancer cells to radiation treatment. Front. Mol. Biosci. *1*, 24.
7. Chen, Z., Li, J.L., Lin, S., Cao, C., Gimbrone, N.T., Yang, R., Fu, D.A., Carper, M.B., Haura, E.B., Schabath, M.B., et al. (2016). cAMP/CREB-regulated LINC00473 marks LKB1-inactivated lung cancer and mediates tumor growth. J. Clin. Invest. *126*, 2267–2279.
8. Lin, S., Zhang, R., An, X., Li, Z., Fang, C., Pan, B., Chen, W., Xu, G., and Han, W. (2019). lncRNA HOXA-AS3 confers cisplatin resistance by interacting with HOXA3 in non-small-cell lung carcinoma cells. Oncogenesis *8*, 60.
9. Hua, Q., Jin, M., Mi, B., Xu, F., Li, T., Zhao, L., Liu, J., and Huang, G. (2019). LINC01123, a c-Myc-activated long non-coding RNA, promotes proliferation and aerobic glycolysis of non-small cell lung cancer through miR-199a-5p/c-Myc axis. J. Hematol. Oncol. *12*, 91.

10. He, J., Wu, K., Guo, C., Zhou, J.K., Pu, W., Deng, Y., Zuo, Y., Zhao, Y., Liu, L., Wei, Y.Q., and Peng, Y. (2018). Long non-coding RNA AFAP1-AS1 plays an oncogenic role in promoting cell migration in non-small cell lung cancer. Cell. Mol. Life Sci. *75*, 4667–4681.

11. Wang, Z.Y., Duan, Y., and Wang, P. (2020). SP1-mediated upregulation of lncRNA SNHG4 functions as a ceRNA for miR-377 to facilitate prostate cancer progression through regulation of ZIC5. J. Cell. Physiol. *235*, 3916–3927.

12. Tang, Y., Wu, L., Zhao, M., Zhao, G., Mao, S., Wang, L., Liu, S., and Wang, X. (2019). *LncRNA SNHG4* promotes the proliferation, migration, invasiveness, and epithelial-mesenchymal transition of lung cancer cells by regulating *miR-98-5p*. Biochem. Cell Biol. *97*, 767–776.

13. Inage, T., Nakajima, T., Itoga, S., Ishige, T., Fujiwara, T., Sakairi, Y., Wada, H., Suzuki, H., Iwata, T., Chiyo, M., et al. (2018). Molecular nodal staging using miRNA expression in lung cancer patients by endobronchial ultrasound-guided transbronchial needle aspiration. Respiration *96*, 267–274.

14. Valencia, K., Erice, O., Kostyrko, K., Hausmann, S., Guruceaga, E., Tathireddy, A., Flores, N.M., Sayles, L.C., Lee, A.G., Fragoso, R., et al. (2020). The *Mir181ab1* cluster promotes *KRAS*-driven oncogenesis and progression in lung and pancreas. J. Clin. Invest. *130*, 1879–1895.

15. Kumar, M.S., Erkeland, S.J., Pester, R.E., Chen, C.Y., Ebert, M.S., Sharp, P.A., and Jacks, T. (2008). Suppression of non-small cell lung tumor development by the *let-7* microRNA family. Proc. Natl. Acad. Sci. USA *105*, 3903–3908.

16. Zhu, W.Y., Luo, B., An, J.Y., He, J.Y., Chen, D.D., Xu, L.Y., Huang, Y.Y., Liu, X.G., Le, H.B., and Zhang, Y.K. (2014). Differential expression of miR-125a-5p and let-7e predicts the progression and prognosis of non-small cell lung cancer. Cancer Invest. *32*, 394–401.

17. Guo, X., and Zhang, Q. (2017). The emerging role of histone demethylases in renal cell carcinoma. J. Kidney Cancer VHL *4*, 1–5.

18. Li, Y., Yang, W., Wu, B., Liu, Y., Li, D., Guo, Y., Fu, H., and Li, Y. (2017). KDM3A promotes inhibitory cytokines secretion by participating in TLR4 regulation of Foxp3 transcription in lung adenocarcinoma cells. Oncol. Lett. *13*, 3529–3537.

19. Ramadoss, S., Guo, G., and Wang, C.Y. (2017). Lysine demethylase KDM3A regulates breast cancer cell invasion and apoptosis by targeting histone and the non-histone protein p53. Oncogene *36*, 47–59.

20. Chuikov, S., Kurash, J.K., Wilson, J.R., Xiao, B., Justin, N., Ivanov, G.S., McKinney, K., Tempst, P., Prives, C., Gamblin, S.J., et al. (2004). Regulation of p53 activity through lysine methylation. Nature *432*, 353–360.

21. Herbst, R.S., Morgensztern, D., and Boshoff, C. (2018). The biology and management of non-small cell lung cancer. Nature *553*, 446–454.

22. Tang, Y., Xiao, G., Chen, Y., and Deng, Y. (2018). lncRNA MALAT1 promotes migration and invasion of non-small-cell lung cancer by targeting miR-206 and activating Akt/mTOR signaling. Anticancer Drugs *29*, 725–735.

23. Lu, Q., Shan, S., Li, Y., Zhu, D., Jin, W., and Ren, T. (2018). Long noncoding RNA *SNHG1* promotes non-small cell lung cancer progression by up-regulating *MTDH* via sponging miR-145-5p. FASEB J. *32*, 3957–3967.

24. Li, H., Hong, J., and Wijayakulathilaka, W.S.M.A. (2019). Long non-coding RNA *SNHG4* promotes cervical cancer progression through regulating c-Met via targeting miR-148a-3p. Cell Cycle *18*, 3313–3324.

25. Jiang, C., Yang, Y., Yang, Y., Guo, L., Huang, J., Liu, X., Wu, C., and Zou, J. (2018). Long noncoding RNA (lncRNA) HOTAIR affects tumorigenesis and metastasis of non-small cell lung cancer by upregulating miR-613. Oncol. Res. *26*, 725–734.

26. Nie, W., Ge, H.J., Yang, X.Q., Sun, X., Huang, H., Tao, X., Chen, W.S., and Li, B. (2016). lncRNA-*UCA1* exerts oncogenic functions in non-small cell lung cancer by targeting miR-193a-3p. Cancer Lett. *371*, 99–106.

27. Zhang, Y.K., Zhu, W.Y., He, J.Y., Chen, D.D., Huang, Y.Y., Le, H.B., and Liu, X.G. (2012). miRNAs expression profiling to distinguish lung squamous-cell carcinoma from adenocarcinoma subtypes. J. Cancer Res. Clin. Oncol. *138*, 1641–1650.

28. Qi, L., Liu, F., Zhang, F., Zhang, S., Lv, L., Bi, Y., and Yu, Y. (2018). lncRNA NEAT1 competes against let-7a to contribute to non-small cell lung cancer proliferation and metastasis. Biomed. Pharmacother. *103*, 1507–1515.

29. Sechler, M., Parrish, J.K., Birks, D.K., and Jedlicka, P. (2017). The histone demethylase KDM3A, and its downstream target MCAM, promote Ewing sarcoma cell migration and metastasis. Oncogene *36*, 4150–4160.

30. Wade, M.A., Jones, D., Wilson, L., Stockley, J., Coffey, K., Robson, C.N., and Gaughan, L. (2015). The histone demethylase enzyme KDM3A is a key estrogen receptor regulator in breast cancer. Nucleic Acids Res. *43*, 196–207.

31. Nie, F.Q., Sun, M., Yang, J.S., Xie, M., Xu, T.P., Xia, R., Liu, Y.W., Liu, X.H., Zhang, E.B., Lu, K.H., and Shu, Y.Q. (2015). Long noncoding RNA ANRIL promotes non-small cell lung cancer cell proliferation and inhibits apoptosis by silencing KLF2 and P21 expression. Mol. Cancer Ther. *14*, 268–277.

32. Chen, Z., Chen, X., Chen, P., Yu, S., Nie, F., Lu, B., Zhang, T., Zhou, Y., Chen, Q., Wei, C., et al. (2017). Long non-coding RNA SNHG20 promotes non-small cell lung cancer cell proliferation and migration by epigenetically silencing of P21 expression. Cell Death Dis. *8*, e3092.

33. Bae, K.M., Wang, H., Jiang, G., Chen, M.G., Lu, L., and Xiao, L. (2007). Protein kinase C*ε* is overexpressed in primary human non-small cell lung cancers and functionally required for proliferation of non-small cell lung cancer cells in a p21/Cip1-dependent manner. Cancer Res. *67*, 6053–6063.

34. Abbas, T., and Dutta, A. (2009). p21 in cancer: intricate networks and multiple activities. Nat. Rev. Cancer *9*, 400–414.

OMTO, Volume *20*

Supplemental information

The long non-coding RNA SNHG4/microRNA-let-7e/KDM3A/p21 pathway is involved in the development of non-small cell lung cancer

Fan Wang and Qingqing Quan

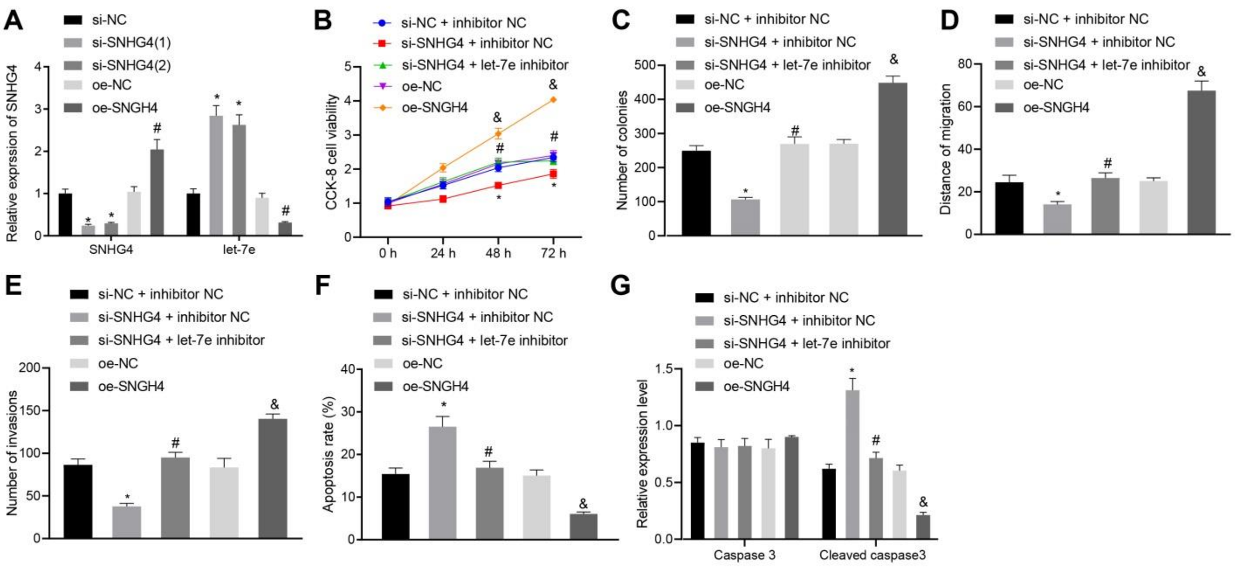

Supplementary Figure 1. LncRNA SNHG4 mediates H1975 cell proliferation, migration, invasion, and apoptosis by binding to miR-let-7e.

A, Expression of lncRNA SNHG4 and miR-let-7e after siRNA knockdown of lncRNA SNHG4 in H1975 cells. B, H1975 cell viability measured by CCK-8 assays. C, Numbers of colonies derived from H1975 cells. D, Scratch wound healing. E, H1975 cells invading from Matrigel-coated upper transwell chambers into lower ones. F, H1975 cell apoptosis determined by Annexin V/PI-labeled flow cytometric analysis. G, Western blot analysis of caspase 3 and cleaved caspase 3 in H1975 cells, normalized to β-actin expression. * (compared with H1975 cells treated with scramble siRNA alone and/or inhibitor NC), # (compared with H1975 cells treated with oe-NC alone or lncRNA SNHG4-specific siRNA plus inhibitor NC) and & (compared with H1975 cells treated with oe-NC alone) indicate $p < 0.05$ by Tukey's test-corrected one-way ANOVA or Bonferroni-corrected repeated measures ANOVA.

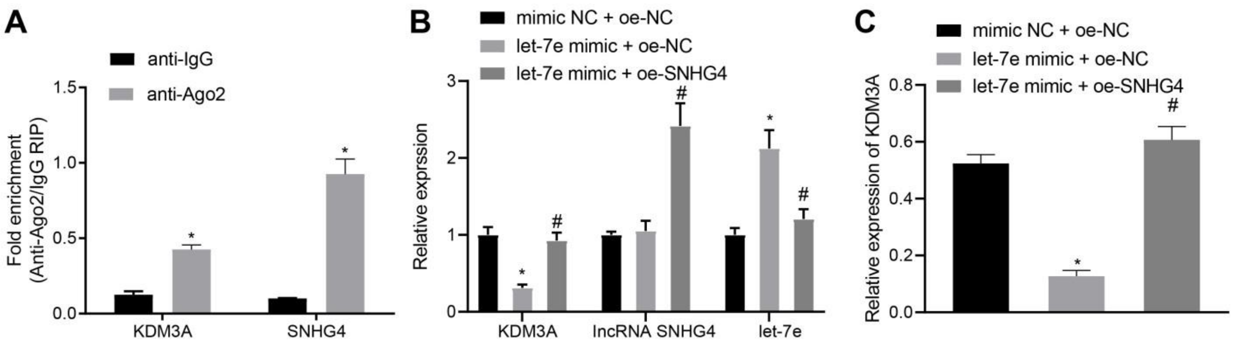

Supplementary Figure 2. LncRNA SNHG4 bound to miR-let-7e and upregulated KDM3A.

A, Anti-Ago2 RIP in H1975 cells transiently overexpressing lncRNA SNHG4. B, The expression of KDM3A was determined by RT-qPCR in H1975 cells. C, Western blot analysis of KDM3A in H1975 cells, normalized to β-actin expression. * (compared with H1975 cells treated with oe-NC with or without mimic NC) and # (compared with H1975 cells treated with miR-let-7e mimic with oe-NC) indicate $p < 0.05$ by unpaired t test or Tukey's test-corrected one-way ANOVA. Pearson Correlation Coefficient was applied to correlation analysis between KDM3A and lncRNA SNHG4.

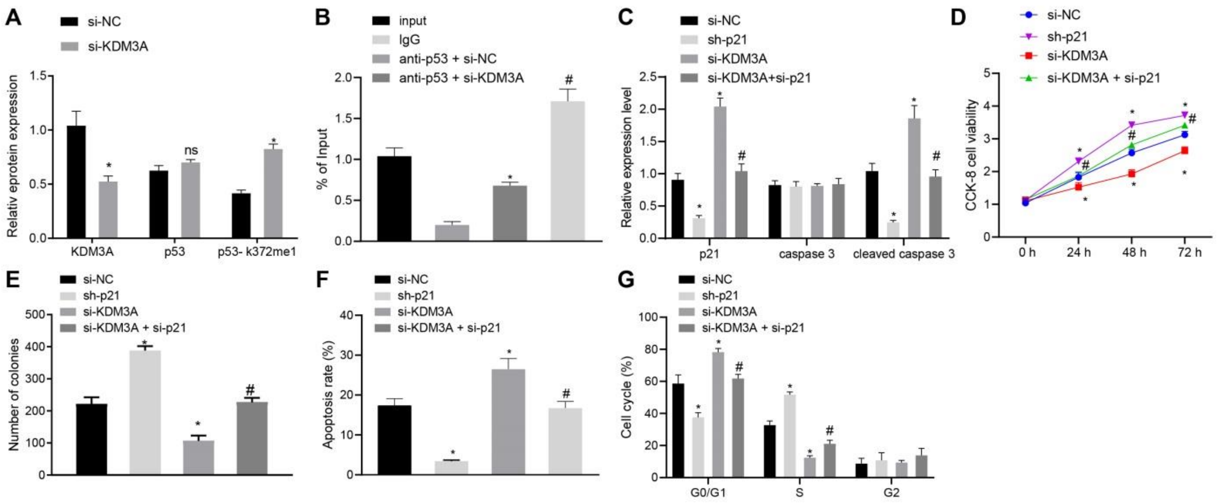

Supplementary Figure 3. KDM3A functioned as an oncogene in NSCLC by inhibiting p21.

A, Western blots and quantification of KDM3A, p53-k372me1, and p53 in H1975 cells, normalized to β-actin expression. B, p21 was immunoprecipitated using p53 antibody relative to IgG by ChIP assays. C, Western blot analysis of p21, cleaved caspase 3, and caspase 3 in H1975 cells, normalized to β-actin expression. D, H1975 cell viability was measured by CCK-8 assays. E, Numbers of colonies derived from H1975 cells. F, H1975 cell apoptosis determined by Annexin V/PI-labeled flow cytometric analysis. G, H1975 cell cycle determined by flow cytometric analysis. * (compared with scramble siRNA or IgG antibody) and # (compared with si-KDM3A or anti-p53 with si-NC) indicate $p < 0.05$ by unpaired t test, Tukey's test-corrected one-way ANOVA or Bonferroni-corrected repeated measures ANOVA.
